# Supplementary material for: A New Investigation into the Molecular Mechanism of Andrographolide towards Reducing Cytokine Storm
Source: Molecules. 2022 Jul 17;27(14):4555. doi: 10.3390/molecules27144555 (PMC9319373; doi:10.3390/molecules27144555)
Supplement: Supplementary file 1 [file molecules-27-04555-s001.zip › molecules-1793052-supplementary.pdf]

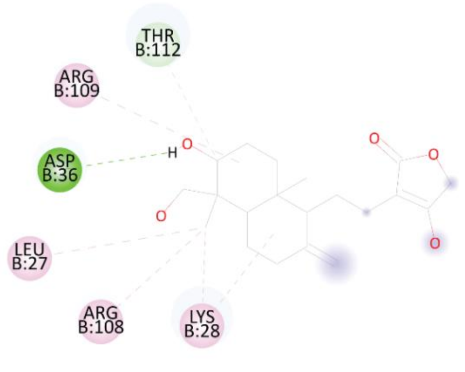

(A)

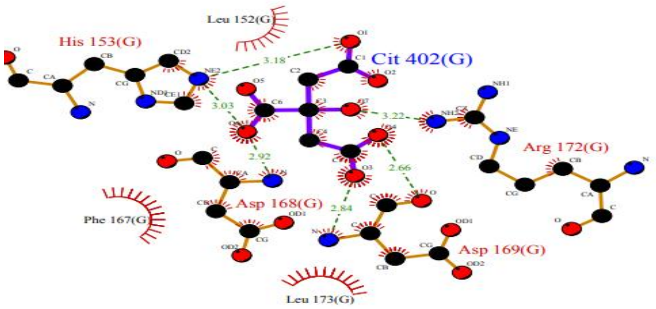

IL-3

COMMON INTETRACTIVE RSSIDURES ARE ASP, ARG WITH SIMILAR INTERACTIVE PAATERN

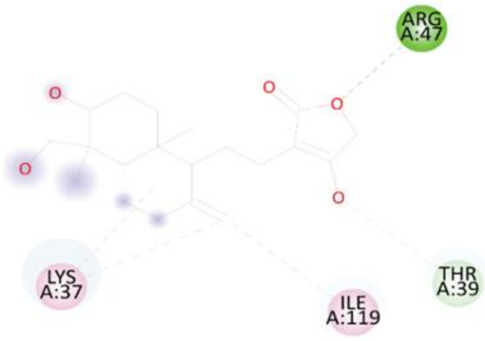

(B)

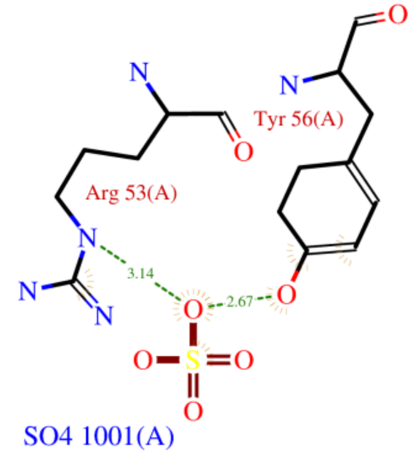

IL-4

COMMON INTETRACTIVE RSSIDURES ARG WITH SIMILAR INTERACTIVE PAATERN

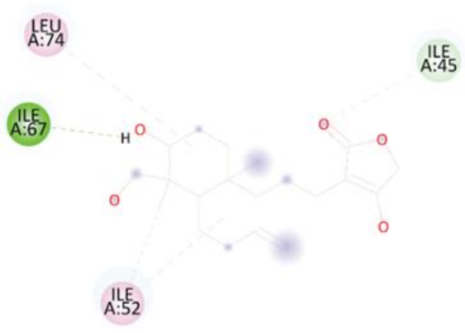

(C)

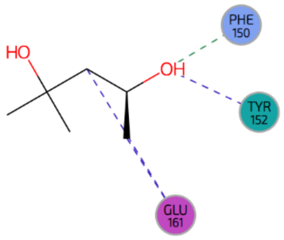

IL-5

NO COMMON INTETRACTIVE RSSIDURES

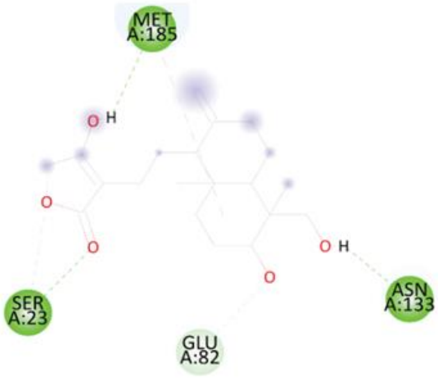

(D)

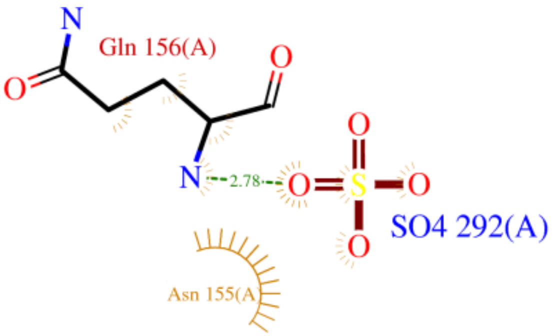

IL-6

COMMON INTETRACTIVE RSSIDURES Asn WITH SIMILAR INTERACTIVE PAATERN

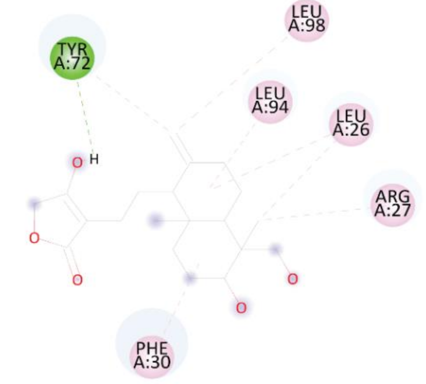

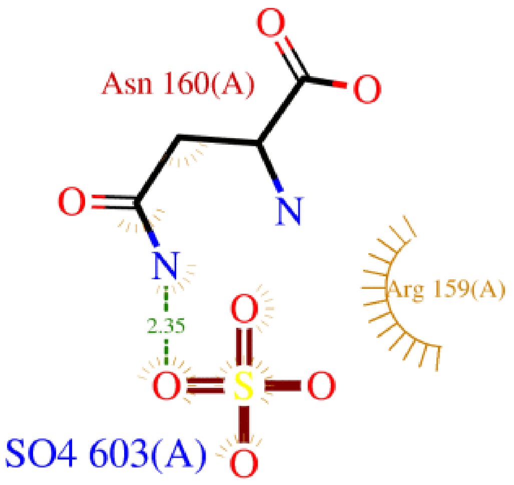

(E)

IL-10

COMMON INTETRACTIVE RSSIDURES Arg WITH SIMILAR INTERACTIVE PAATERN

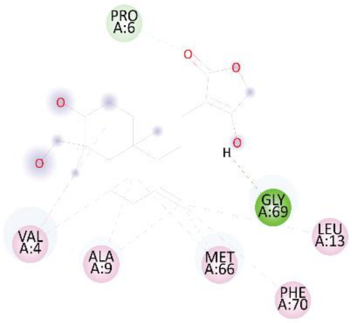

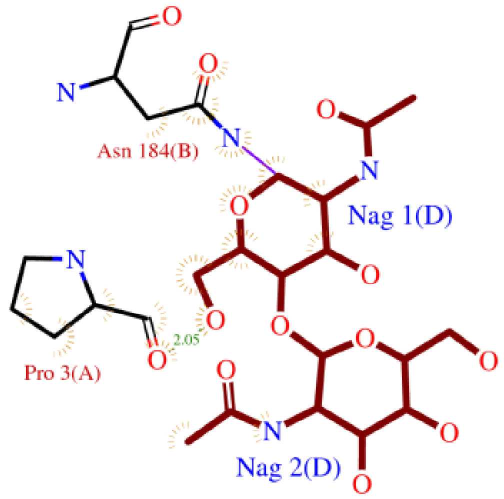

(A)

IL-13

COMMON INTETRACTIVE RSSIDURES PRO WITH SIMILAR INTERACTIVE PAATERN

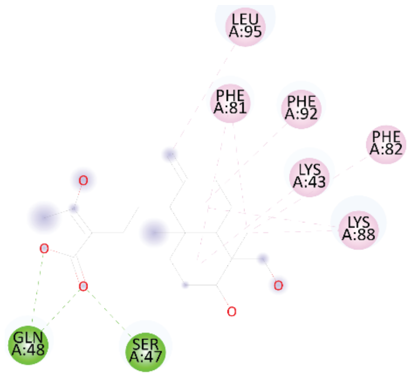

(B)

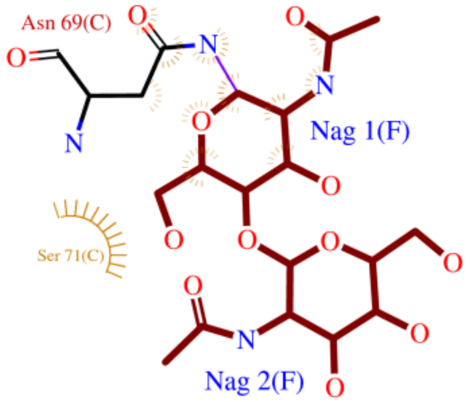

IFN-γ

COMMON INTETRACTIVE RSSIDURES SER WITH SIMILAR INTERACTIVE PAATERN

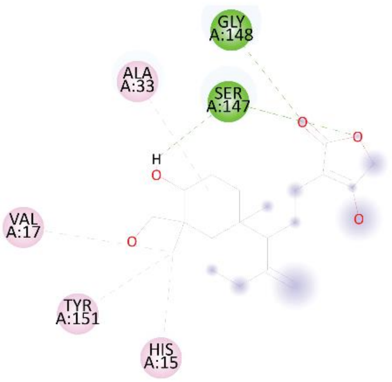

(C)

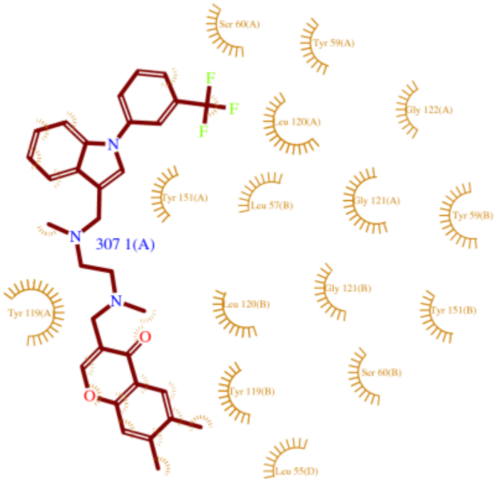

TNF-a

COMMON INTETRACTIVE RSSIDURES SER and Gly WITH SIMILAR INTERACTIVE PAATERN

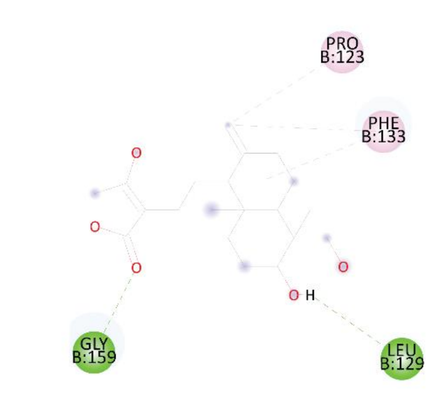

(D)

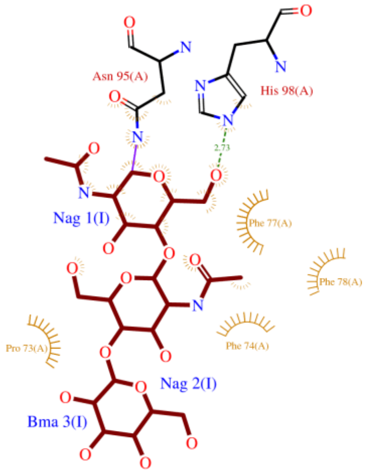

G-CSF

COMMON INTETRACTIVE RSSIDURES PRO and PHE WITH SIMILAR INTERACTIVE PAATERN

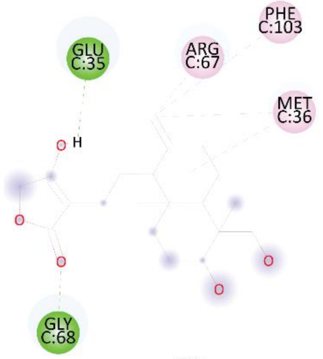

(E)

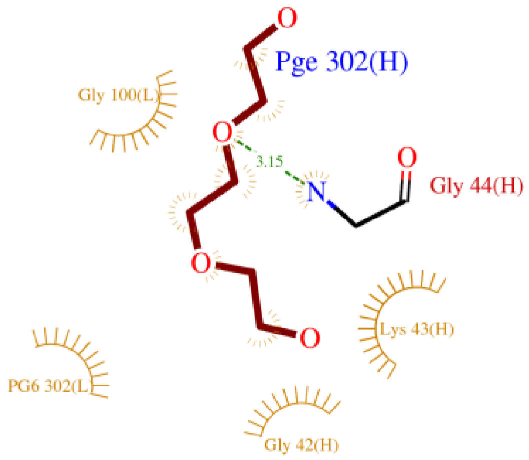

GM-CSF

COMMON INTETRACTIVE RSSIDURES Gly WITH SIMILAR INTERACTIVE PAATERN

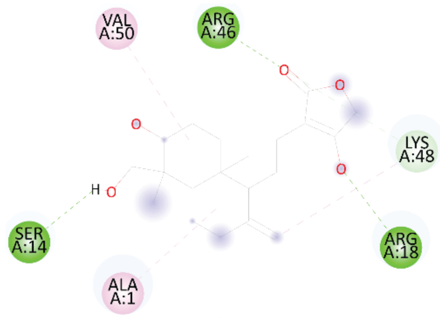

(F)

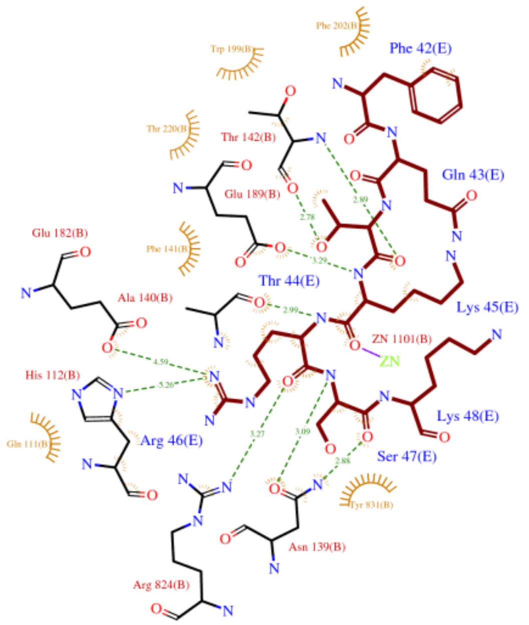

MiP1B

COMMON INTETRACTIVE RSSIDURES Arg,Ser and Lys WITH SIMILAR INTERACTIVE PAATERN

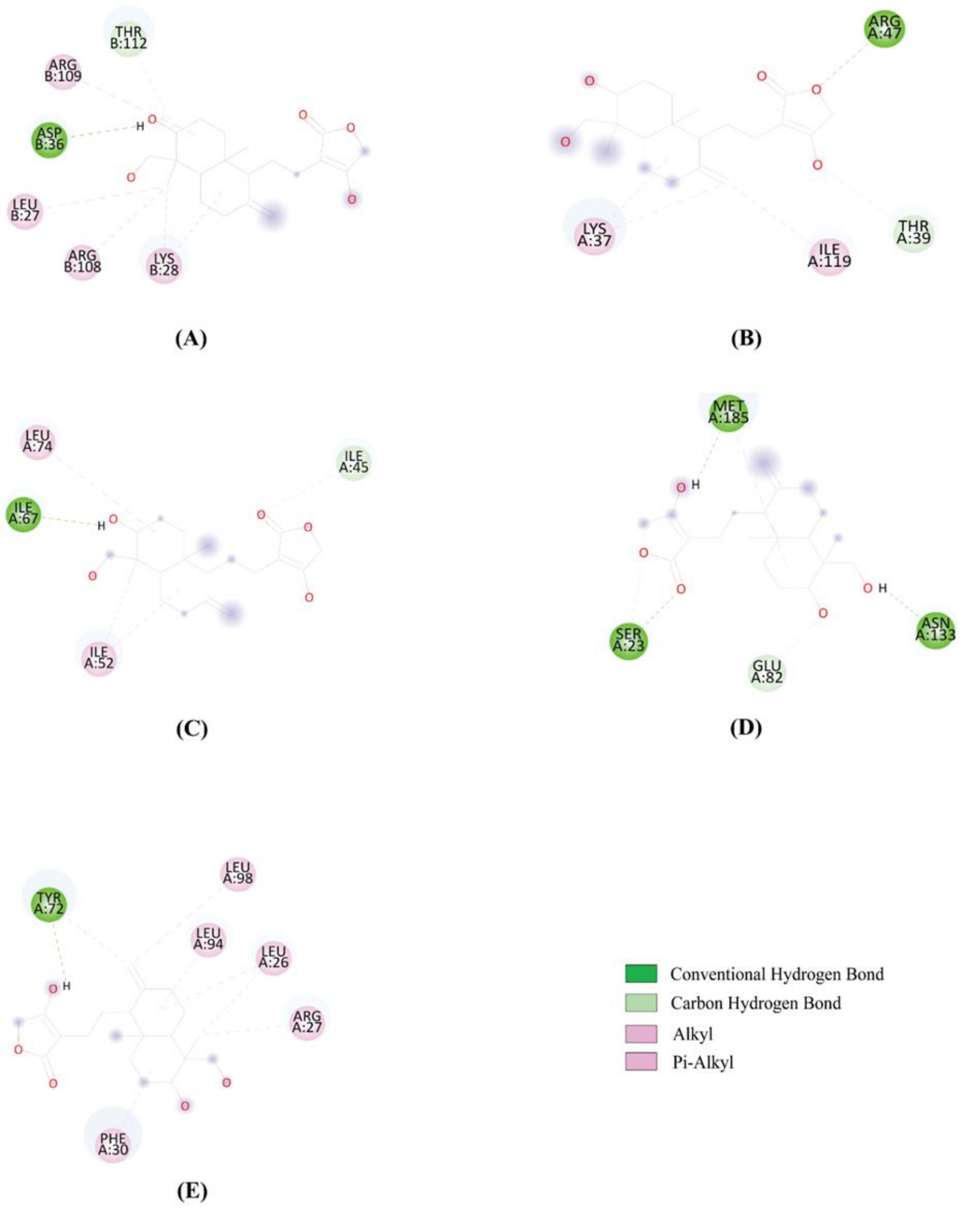

Interactions of IL-3 (**A**), IL-4 (**B**), IL-5 (**C**), IL-6 (**D**) and IL-10 (**E**) with Andrographolide.

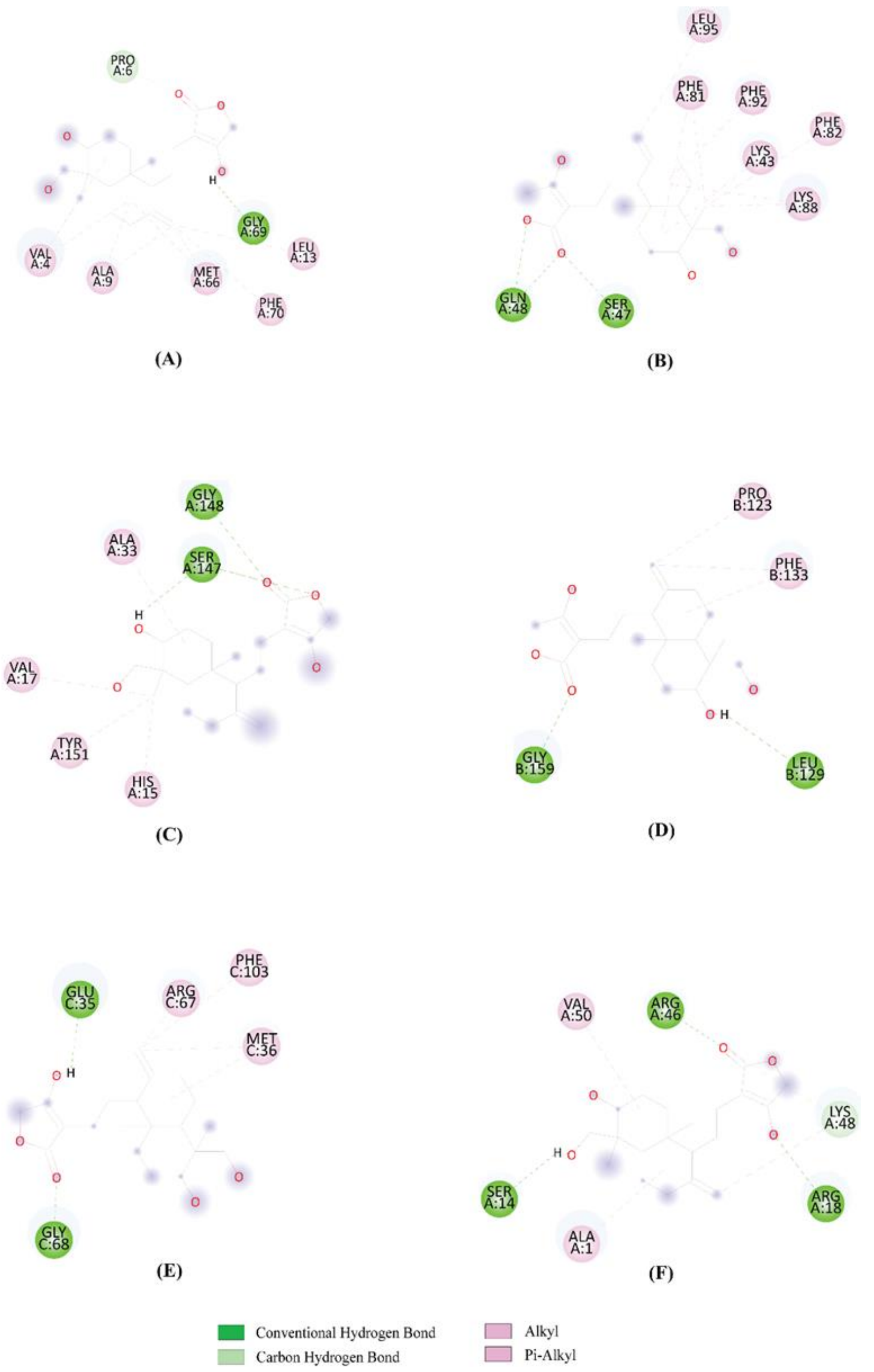

Interaction of IL-13 (**A**), IFN-γ (**B**), TNF-α (**C**), G-CSF (**D**), GM-CSF (**E**) and MiP1β (**F**) with Andrographolide.

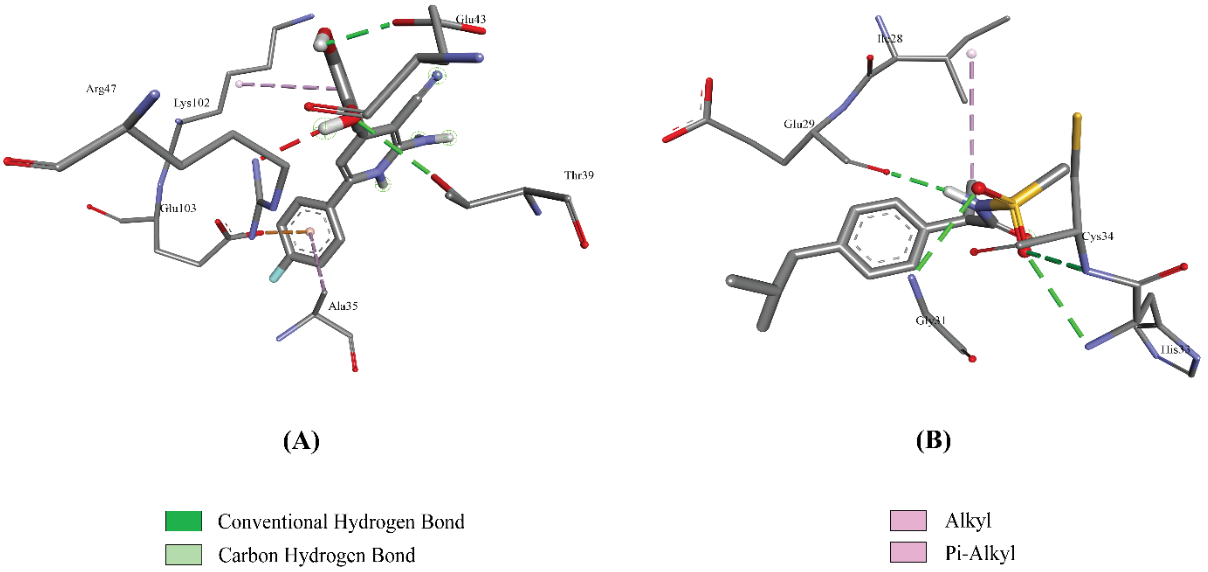

Interactions of control compounds with (A) IL-4 and (B) IL-8
